# Overview of Dry Eye Disease for Primary Care Physicians

**DOI:** 10.3390/medicina61030460

**Published:** 2025-03-06

**Authors:** Jeonghyun Kwon, Amirhossein Moghtader, Christie Kang, Zahra Bibak Bejandi, Sumaiya Shahjahan, Ahmad Alzein, Ali R. Djalilian

**Affiliations:** 1 Department of Ophthalmology and Visual Sciences, University of Illinois at Chicago, Chicago, IL 60612, USA; kwon16@uic.edu (J.K.); moghtada@uic.edu (A.M.); zhrbibak@uic.edu (Z.B.B.); sumaiya.shahjahan@dmu.edu (S.S.); aalzei2@uic.edu (A.A.); 2 Department of Physiology and Biophysics, University of Illinois at Chicago, Chicago, IL 60612, USA; ckang30@uic.edu

**Keywords:** dry eye disease, keratoconjunctivitis sicca, aqueous-deficient dry eye, evaporative dry eye, ocular surface, cornea, primary care, Sjögren syndrome, meibomian gland dysfunction

## Abstract

Dry eye disease (DED), also known as keratoconjunctivitis sicca, is a multifactorial ocular disease characterized by tear film insufficiency due to diverse etiologies including aging, incomplete and infrequent blinking, hormonal changes, medications, and systemic diseases. Classified into aqueous-deficient dry eye (ADDE), evaporative dry eye (EDE), and mixed subtypes, DED presents with symptoms such as irritation, stinging, redness, foreign body sensation, sensitivity to light, and blurred or fluctuating vision. While rare, severe cases may lead to vision loss. With its rising global prevalence across age groups, DED poses a significant public health challenge. Primary care physicians (PCPs), often the first point of contact for DED patients, require timely screening and management strategies. This review explores the epidemiology, pathophysiology, clinical manifestations, diagnosis, and management of DED, emphasizing practical approaches for PCPs. This narrative review was conducted by searching MEDLINE, PubMed, and Google Scholar databases for relevant articles. Diagnostic approaches, including detailed history taking, patient-reported questionnaires, differential diagnosis, and assessments are discussed alongside management strategies, including symptomatic ophthalmic treatment, risk factor mitigation (e.g., reduced digital device screen time), prevention, and nutrition. By providing a synopsis of early symptoms that PCPs are often the first to encounter, practical approaches to screening and managing DED in the primary care setting, and guidelines on when to refer to specialty care, this comprehensive review aims to equip PCPs with the knowledge to improve DED screening and optimize patient outcomes.

## 1. Introduction

DED is an ocular condition that impacts both the quality and quantity of the tear film, leading to symptoms such as eye discomfort and vision problems [1]. If left untreated, DED holds the risk for long-term consequences such as chronic inflammation leading to tissue damage and scarring in the visual axis and ultimately visual impairment. As the first line in healthcare, PCPs have the opportunity to be the first to encounter and recognize DED in a clinical setting. However, diagnostic barriers and a lack of clear guidelines persist for PCPs in tackling DED. To address these gaps, this review provides practical approaches that equip the PCP to potentially screen, assess the severity of, and manage DED, while knowing when to refer patients for specialized care. This review aims to offer an overview of DED, developed from searching the keywords “dry eye syndrome” and “keratoconjunctivitis sicca” in MEDLINE, PubMed, and Google Scholar databases, with a focus on how non-specialists should approach managing DED. This review incorporates Tear Film and Ocular Surface Society (TFOS) Dry Eye Workshop (DEWS) II reports in simpler language. PCPs can utilize the information in this review to make decisions in diagnoses, management, and referrals regarding their patients’ ocular health.

## 2. Epidemiology

Determining the accurate prevalence of DED globally remains a challenge due to the heterogeneity of definitions, diagnostic tests, and qualifying criteria for DED. The prevalence of DED in Africa is reported to be 42%, with no sex-dependent differences reported [2]. In Asia, the overall prevalence is observed to be 20.1%, with a higher prevalence of 21.7% reported for female patients than the 16.7% prevalence reported for male patients. A general trend of prevalence of DED increasing with age was also reported [3]. In the United States, the prevalence of DED was estimated to be 8.1% [4]. It is difficult to attribute the differences in prevalences observed across various studies to population-specific differences or criteria-dependent differences. Thus, a wide range of prevalences are found to be reported in the literature [5].

To address these discrepancies and better understand the overall impact of DED, Papas describes the usage of a Bayesian approach to model global prevalence of DED using a beta distribution. Analysis of data from multiple studies from 1997 to 2021 using this approach produced an estimate of 11.59% for the global prevalence of DED. While this provides a birds-eye view of DED, this number and analysis is limited by the inclusion criteria of the primary studies, which are heavily biased by the individual differences and criteria set by different research groups [6].

Similarly, a cross-sectional study of interviews conducted across Spain showed different prevalence rates, depending on the criteria that was used to define DED. According to criteria set forth by the Women’s Health Study, 16.6% of the general population was found to suffer from DED. However, when using the Beijing Eye Study definition instead, prevalence seemed to jump to 22.5% [7]. This difference within the same study highlights how different criteria can influence the measured and reported prevalence of DED.

Despite these differences, it is widely accepted that symptoms of DED are reported to increase linearly with age, particularly after the age of 40 [5]. However, strikingly, the prevalence of DED in school children is not nonsignificant. In African school population-based studies, the prevalence rate of DED is as high as 51.6% [2]. In high school students in the Shandong province of China, 23.7% of high school students were clinically diagnosed or exhibited severe symptoms of DED [8]. Similarly, over 20% of Japanese high school students reported severe symptoms of DED [9]. Risk factors that contribute to this unusually high prevalence in younger populations are speculated to include the increase in screen time. With the rising interest in DED in young populations, a review amalgamated some of these findings with other studies around the globe to reveal a range of 5.5 to 26.6% of children affected by DED. Due to the differing study methodologies and criteria, these differences could be attributed to multiple factors. The overall role of ethnicity in attributing to DED in children was inconclusive from this review, but it was noted that Asian children had higher rates of DED than Caucasian children in New Zealand [10,11]. Several studies provide arguments for digital screen time as a significant culprit [12,13,14]. However, it is important to consider that these deleterious effects may be influenced by confounding variables such as mental health disorders, lack of sleep, and sedentary lifestyles [5].

More studies are warranted to definitively define the risk factors of DED. However, a few risk factors have been well substantiated across multiple studies. Increased age and female sex are associated with higher prevalences of DED. Asian ethnicity was also reported in several studies to be a risk factor for DED. Modifiable lifestyle factors that increase the risk of DED include contact lens wear, daily screen time, psychological stress, antidepressant medication, and oral contraceptive therapy [15,16,17].

## 3. Pathophysiology and Etiology

### 3.1. Pathophysiology

**Figure 1:**
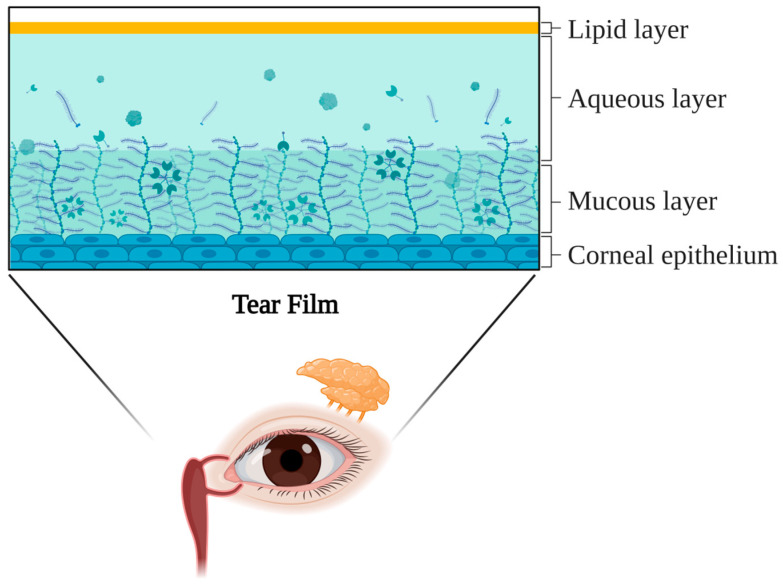
The three-layer model of the tear film. The proportion of the depicted three layers is not representative of the actual composition of the tear film. The mucin layer thickness is exaggerated for the purpose of depicting the different layers.

Among the ocular surface components that maintain the refractive quality, the tear film is the most dynamic [18]. A stable tear film is crucial for ocular health, providing corneal lubrication and serving as the primary refractive surface [19]. Traditionally, the tear film has been described as having three distinct layers (Figure 1): a lipid layer that prevents evaporation, an aqueous layer that supplies lubrication and nutrients, and mucin layers that anchor the hydrophilic aqueous layer to hydrophobic epithelial cells [20]. More recently, a two-layer model has gained acceptance, which consists of a lipid layer overlying a mucoaqueous layer that makes up the majority of the tear film [19]. The lacrimal glands produce most of the tear volume, while the meibomian glands secrete meibum to form the lipid layer [19].

Disruption of the tear film can lead to DED. DED is defined as “a multifactorial disease of the ocular surface characterized by a loss of homeostasis of the tear film, and accompanied by ocular symptoms, in which tear film stability and hyperosmolarity, ocular surface inflammation and damage, and neurosensory abnormalities play etiological roles [1] (p. 277)”. The hallmark of the underlying pathophysiology of DED is evaporation-induced tear hyperosmolarity [1,21]. This hyperosmolarity damages the underlying tissues by inducing inflammation or direct mechanical abrasion, particularly of the epithelial and goblet cells [21], which further exacerbates DED.

### 3.2. Etiology

This review structures the etiologies of DED according to the TFOS DEWS II classification [1,21]. DED is broadly categorized into aqueous deficiency dry eye (ADDE) and evaporative dry eye (EDE), although they exist on a spectrum with a mixed DED category [22]. In fact, the most common phenotype is the mixed type.

#### 3.2.1. ADDE

In ADDE, tear hyperosmolarity occurs primarily due to the decreased amount of tear production. ADDE is subdivided into Sjögren’s Syndrome Dry Eye (SSDE) and Non-Sjögren Syndrome Dry Eye (NSDE) [21].

- SSDE: In Sjögren’s syndrome, inflammatory cells infiltrate the lacrimal gland epithelial cells, leading to reduced tear production and a decreased number of conjunctival goblet cells [21,23]. As a result, SSDE patients have not only reduced tear volume but also increased inflammatory markers (IL-1a, IL-6, IL-8, and TNF-a) [24]. Several other systemic diseases that are associated with SSDE include but are not limited to rheumatoid arthritis, systemic lupus erythematosus, systemic sclerosis, and mixed connective tissue diseases [21].
- NSDE: Other conditions that can decrease the aqueous portion of the tear film include the following: aging, lacrimal gland deficiency/inflammation, lacrimal gland duct obstruction from cicatricial changes, reduced lacrimal gland reflex from neurotrophic changes (corneal nerve damage from refractive surgery, or diabetes mellitus; sensory impulse damage from multiple sclerosis), and certain medications (antihistamines, beta blockers, diuretics, etc.) [8,21,25,26,27].

#### 3.2.2. EDE

While both ADDE and EDE present with excessive tear evaporation, EDE is primarily characterized by tear hyperosmolarity that persists despite normal lacrimal secretion. EDE is further categorized as intrinsic or extrinsic EDE [1].

- Intrinsic EDE: EDE arises from intrinsic ocular causes that directly contribute to the evaporative loss from the tear film [1,15]. Meibomian glands directly contribute to the lipid layer of the tear film. However, in meibomian gland dysfunction (MGD), the subsequent decrease in the lipid component of the tear film leaves the eye more susceptible to excessive tear evaporation [21]. Another potential component of intrinsic EDE is irregularity of the eyelid, which is critical for maintaining the proper closure of eyelids. Likewise, infrequent blinking, which is often found in the elderly, is another common cause of DED. Diseases, such as Parkinson’s disease, that disrupt the function of blinking, can also contribute to EDE [28]. In thyroid eye disease (TED), DED can develop from exophthalmos, which increases the surface area of exposure and subjects the tear film to excessive evaporation [29]. It is noteworthy that both hypothyroidism and hyperthyroidism can cause DED. Therefore, it is reasonable to perform thyroid disorder screening for patients with persistent dry eyes.
- Extrinsic EDE: Extrinsic causes of EDE are non-ocular conditions that disrupt the ocular surface such as contact lens use or preservatives in eyedrops [21]. In particular, the mechanical friction caused by contact lenses destroys goblet cells and decreases mucin secretion subsequently [30,31]. Systemic conditions can also degrade the ocular surface. Xerophthalmia, which stems from a lack of vitamin A, is hypothesized to interfere with mucin synthesis [32]. Androgens are important promoters of the mucin layer of the tear film by regulating the lacrimal and Meibomian glands. Thus, the differing androgen production in biological males and females may explain the sex-dependent DED prevalence differences [33]. While EDE can arise from various factors affecting the eyelid or ocular surface, in young adults, prolonged screen time and long working hours in dry environments may be indicative of an EDE etiology [34,35].

## 4. Clinical Manifestations

Ocular discomfort (i.e., dryness, redness, gritty sensation, pain, photosensitivity) is the hallmark of clinical presentation of DED, with patients also frequently reporting symptoms of foreign body sensation, irritation, fluctuating vision, or blurred vision that are temporarily relieved with blinking or artificial tears [22,36,37]. These symptoms are typically worse later in the day [38] because of prolonged ocular surface exposure to the air and reduced tear film stability [39]. Symptoms could vary based on the severity of DED). Mild symptoms typically include a sandy or gritty sensation, burning, redness, and excessive blinking; moderate cases involve eye pain, fatigue eyes, eyelid twitching, and difficulty keeping the eyes open [40]. Severe DED can lead to debilitating symptoms such as vision fluctuation, photophobia, and even difficulty crying or reading [40]. Its progression may lead to complications such as ulceration, corneal perforation, scarring, and subsequent vision loss in advanced cases [25,36,40]. It is important to note that DED exists on a spectrum, and there is often overlap in patient presentation in mild, moderate and severe DED. Likewise, the signs and symptoms based on the different etiologies of DED—ADDE and EDE—may slightly differ; However, the most common type is the mixed type, and both moderate and severe DED are treated for both mechanisms regardless. DED’s course of action is often considered aggravating in its nature regardless of intervention. This notion was challenged by a retrospective study in which participants with a positive diagnosis or severe DED were included. This study reported that the average duration of DED is 10.5 years for men and 14.5 years for women [5].

DED is typically bilateral but sometimes unilateral and asymmetric in severity when unilateral neurotrophic alterations occur, including unilateral infection or intracranial mass [22]. Common ocular signs include tear film instability, tear hyperosmolarity, reduced tear volume, superficial corneal erosions, conjunctival hyperemia, and MGD [36]. Counterintuitively, DED can cause tearing because eye irritation may cause reflex tearing, and this can mask dry eye symptoms [22].

DED symptoms can manifest, especially after refractive surgery, with the following symptoms: tear dysfunction, neurotrophic epitheliopathy, and corneal sensitivity changes [41]. Symptoms are more severe and longer lasting after laser-assisted in situ keratomileusis (LASIK) compared to small incision lenticule extraction (SMILE) or photorefractive keratectomy (PRK) [41]. Ocular surface health is critical in mitigating the risk and severity of DED following anterior segment surgeries (e.g., refractive surgery, cataract surgery). DED symptoms can also manifest secondary to psychological disorders such as depression, sleep disorders, and mood disorder; neurological disorders such as migraines; and metabolic disorders including dyslipidemia [25].

A critical challenge of DED is its poor correlation between symptoms and clinical signs, complicating diagnosis and management. Some patients experience significant discomfort with minimal clinical signs, while others with severe dry eye complications may report only mild symptoms [42]. In 2017, experts categorized the signs and symptoms of DED based on general manifestations, various pathophysiology (EDE and ADDE), and differential diagnosis of DED [43]. Notably, they found that clinical signs were more reliable than patient-reported symptoms and history in distinguishing DED etiologies [43]. Similarly, Donthineni et al. also stated that symptoms alone were not predictive of different types of DED [44].

## 5. Diagnosis

A careful and thorough approach to diagnosing DED is critical, as it not only facilitates proper management but also minimizes the risk of misdiagnosis and subsequent interventions that can exacerbate the condition. The complexity of diagnosing DED arises from its multifactorial nature and variable clinical presentations across patients [45]. DED, as a result, is diagnosed by integrating the history of present illness, physical examination findings, and diagnostic test results [26]. TFOS DEWS II Diagnostic Methodology Report presents a clinical protocol for DED diagnosis. The protocol begins with triaging questions to identify risk factors and to rule out mimicking diseases. Then, if the Dry Eye Questionnaire (DEQ-5) scores ≥ 6 or Ocular Surface Disease Index (OSDI) ≥ 13, one positive result of any of the following homeostasis tests is required for a DED diagnosis: Non-Invasive Breakup Time (NIBUT) < 10 s, ≥308 mOsm/L osmolarity in at least one eye or >8 mOsm/L difference between the eyes, and ocular surface staining > 5 corneal spots, >9 conjunctival spots, or ≥2 mm length and ≥25% width of lid margin [46].

The suggested diagnostic homeostasis tests require specialist skill. However, PCPs can screen and determine the need for referral by (1) gathering a thorough history of present illness, (2) establishing a differential diagnosis, and (3) performing a few simple ancillary tests. As the first point of contact for patients, PCPs are well positioned to promptly screen DED-associated symptoms and initiate symptomatic treatment via basic ophthalmic management [47].

### 5.1. Step 1: Gather Thorough History of Present Illness

Obtaining a detailed patient history is fundamental to diagnosing DED [36]. Both ocular and non-ocular symptoms of DED should be assessed, along with the risk factors including occupational and environmental exposure. A patient’s ocular history and systemic past medical history associated with DED can provide important diagnostic clues. A thorough medication review is also critical, as topical eyedrops containing preservatives or certain systemic medications such as antihistamines, diuretics, etc. may contribute to dry eye symptoms [38,42]. Certain aspects of medical history and their individual risk factor profiles should be closely evaluated to assess the need for further workup or a referral. Patients with comorbidities, such as autoimmune diseases and systemic inflammatory diseases, require close attention during screening because of the high association between their diseases and DED (Table 1). For instance, PCPs should take extra care to not miss manifestation of DED in a patient with Sjögren’s syndrome and should promptly refer the patient to an eye specialist for further workup.

Validated DED questionnaires play an essential role in eliciting pertinent history and quantifying the symptoms.

- OSDI: The OSDI is a widely used questionnaire designed to evaluate dry eye symptoms and their effect on quality of life over the past one week [52]. It comprises 12 questions, each rated on a scale from “none of the time” (0 points) to “all of the time” (4 points) (Table 2). The final OSDI score is calculated as Equation (1).
  (1) $$Final\;OSDI\;Score\;=\;\left(\frac{Sum\;of\;Points\;\times \;25}{Total\;Number\;of\;Answered\;Questions}\right)$$
  On the OSDI scale, the severity of DED is defined as mild (13–22), moderate (23–32), and severe (>33) [52,53]. While the OSDI assesses the symptom severity and visual function, it does not fully capture the daily burden of DED or monitor the treatment effects [54].
- DEQ-5: The DEQ-5 has five questions on the frequency, intensity, and discomfort of DED symptoms (Table 3). A score of >6 indicates the presence of DED, while a score of >12 warrants further investigation for potential DED secondary to Sjögren’s Syndrome [55].

### 5.2. Step 2: Establish a Differential Diagnosis

In cases of severe or unilateral presentations, or when suspected DED symptoms persist despite more than one month of treatment in non-specialist care settings, a thorough differential diagnosis and detailed eye examination are paramount to ensure accurate identification of DED [45,46]. PCPs are in an advantageous position to ask differential diagnosis questions that address various/holistic aspects such as lifestyle (working in dry environments), history of autoimmune/systemic diseases, activities (swimming in open water), iatrogenic causes (medications), exposure to sick contacts, and any coexisting ocular conditions to accurately identify DED and the necessary treatments. Differentials for DED include conjunctivitis (allergic, bacterial, and viral), anterior blepharitis, parasitic infections, autoimmune conditions, mood disorders, and various corneal and conjunctival diseases [46]. These conditions may contribute to its development, coexist with DED, or arise as a secondary consequence. When the secondary causes of EDE—including rosacea, psoriasis, Sjögren syndrome, graft-versus-host-disease, rheumatoid arthritis, systemic lupus erythematous, Stevens-Johnson syndrome, sarcoidosis, scleroderma, lymphoma, sarcoidosis [38], are suspected, pertinent systemic physical examinations should be pursued. For example, common dermatologic etiologies of MGD are acne rosacea, atopic dermatitis, and psoriasis. PCPs can look for pertinent skin examination findings and treat the underlying causes.

### 5.3. Step 3: Perform Exams and Ancillary Tests in Office

Once DED is suspected from the patient history and DED questionnaires, PCPs can perform several simple physical exams and ancillary tests in the office to screen for DED. Nevertheless, it is imperative to acknowledge that all of the test results presented in this review should be interpreted in the context of each individual patients, considering the multifactorial nature of DED [56].

**Figure 2:**
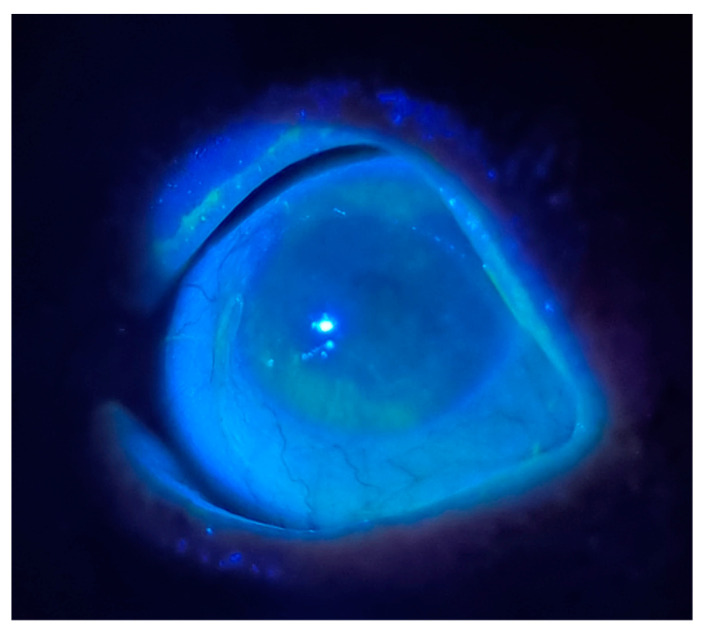
Gross examination of DED in 68-year-old female patient with Sjögren’s syndrome and rheumatoid arthritis. Fluorescein was applied on lower conjunctival sac and blue light from ophthalmoscope was illuminated in dark room to visualize corneal damage.

- Blinking: Patients with DED often exhibit increased blinking frequency compared to those without the condition. A study reported a mean blink interval of 5.97 s in normal subjects versus 2.56 s in those with DED [57]. The Optrex^TM^ Dry Eye Blink Test is an online self-assessment where patients look at the screen without blinking and measure how long it takes for them to feel discomfort [58]. The results showed negative correlations with OSDI score (*p* = 0.006), DEQ-5 score (*p* = 0.004), conjunctival staining score (*p* = 0.03), and inferior lid wiper epitheliopathy grade (*p* = 0.02); the Blink Test demonstrated its diagnostic ability with 66% sensitivity and 88% specificity (*p* < 0.0001) [58].
- Fluorescein Dye: Fluorescein sodium may be applied to the corneal surface to visualize abrasions and DED. It can be applied via a drop (2 µL of a 1% solution) or pre-prepared fluorescein-stained strip. When visualized under cobalt-blue light, fluorescein-stained epithelium will shine bright green [59]. PCPs can accomplish this exam using the blue light from the ophthalmoscope (Figure 2). Visualization of the disrupted integrity of the tear film and damage to the corneal epithelium is particularly useful in assessing DED (Figure 2).
- Eyelid Structure: Although a microscopic eyelid examination is not feasible in the primary care settings, certain structural and functional abnormalities associated with DED can be identified grossly with the naked eye [60]. These include entropion (inward turning of the eyelid), ectropion (outward turning of the eyelid), incomplete eyelid closure, and trichiasis (eyelashes touching the ocular surface).
- Schirmer Test: A simple test strip may be placed in the inferior cul-de-sac for five minutes and used to measure the length of the tear mark on the strip. A tear length of ≤10 mm is considered abnormal. The Schirmer test without anesthesia measures the basal and reflex tearing, while testing with anesthesia only measures basal tear production. Although the Schirmer test is not to be used in isolation to diagnose DED [61], consistently low results across serial Schirmer tests strongly indicate ADDE [38]. The Schirmer test without anesthesia is also most effective in identifying patients with severe dry eyes; however, its variability and limited sensitivity make it less reliable for detecting mild to moderate cases [62].
- Laboratory Tests: Matrix metalloproteinase-9 (MMP-9), an inflammatory marker, is elevated in severe DED. When DED is secondary to a systemic condition, additional laboratory tests can help identifying the etiology of DED: Anti-Ro, anti-La, ANA (Sjögren syndrome); rheumatoid factor (rheumatoid arthritis); antithyroid peroxidase antibody and antithyroglobulin antibody (TED); and serum lysozyme and ACE (sarcoidosis) [38].
- Corneal Sensation: Researchers indicate that DED can heighten corneal sensitivity, as the disrupted tear film and desquamated epithelium expose the corneal nerves to external stimuli [63,64]. Others argue that corneal sensation decreases due to the morphological damage in corneal nerve endings [65,66]. Regardless, the dysfunctional tear film and hyperosmolarity of DED increase the risk for abnormal corneal sensation. Corneal sensation is traditionally assessed using the Cochet-Bonnet esthesiometer, which quantifies the sensitivity based on the length of microfilament that triggers a response [38]. However, in primary care settings, corneal sensation can be qualitatively assessed with a cotton tip. First, a cotton tip needs to be manually displaced into fine wisps and used to assess the central and peripheral corneal sensation with a grading of “absent”, “decreased”, “normal”, and “increased” [67,68,69]. Patients with neurotrophic keratitis—which manifests with decreased corneal sensation in the setting of trigeminal nerve damage, diabetes, or viral infection [69]—would have a diminished sense of pain from DED. Comorbidity of DED in this patient population can worsen the progression of neurotrophic keratitis [67], and DED assessment becomes crucial to prevent corneal complications.

### 5.4. Diagnostic Challenges and Referral to Specialists

There are inconsistencies between symptoms and signs, as noted in the clinical presentation section of this review. This lack of consistency poses a barrier for PCPs to correctly identify and screen for DED, which brings to attention the need for more specific DED guidelines and well-defined diagnostic criteria of clinical tests. This heterogeneous symptom spectrum warrants training of PCPs on the various symptomology of DED. A study showed statistically significant differences between PCPs and optometrists in the identification of symptoms that were associated with DED [61]. PCPs had a shorter list of symptoms, and the ocular symptoms that PCPs missed to associate with DED were itching, temporary blurred vision, eyelid adhesion in the morning, ocular and periocular pain, photophobia, eyelid and conjunctival redness, and eye strain with DED [61]. Research also showed that PCPs infrequently use objective DED diagnostic tests, which was speculated to be due to limited access to specialized equipment [61].

**Figure 3:**
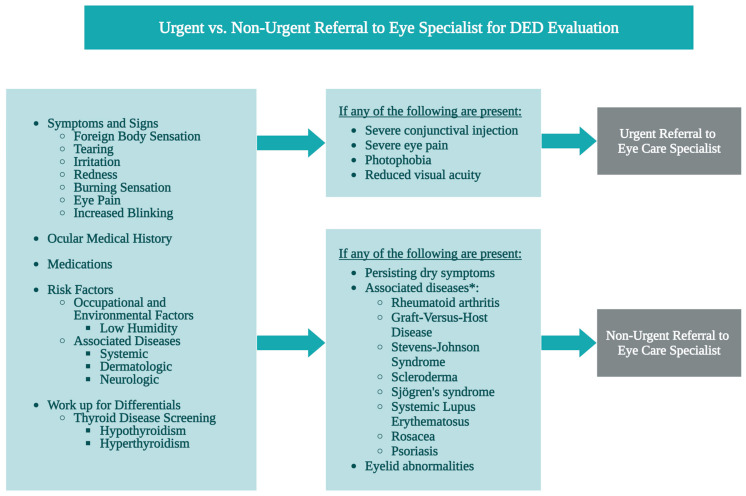
An overview of DED diagnostic tests for PCPs and indicators for non-urgent and urgent referrals to an eye care specialist. * The associated diseases listed on Figure 3 are not exhaustive of all possible autoimmune diseases.

There are alarming indications for referral for a full diagnosis and assessment by an eye care specialist (i.e., an optometrist or an ophthalmologist) (Figure 3). The National Institute for Health and Care Excellence (NICE) guidelines list five indications for referral. When any of the following criteria is met, a PCP should make a referral [45]:

1. Moderate–severe eye pain, photophobia, marked redness in one eye or reduced visual acuity.
2. Worsening vision.
3. Ulcers or corneal damage signs.
4. Persisting or worsening symptoms despite treatment for 4 weeks.
5. Associated disease requiring specialist treatment.

### 5.5. Slit Lamp Examination by Ophthalmologist or Optometrist

When patients are referred to eye specialists, a slit lamp examination allows for a more detailed evaluation of the eye. Several in-depth physical examinations and diagnostic tests can be performed using this specialized equipment for DED diagnosis, including the following:

- NIBUT: The stability of the tear film can be quantified by measuring how long it takes for it to break up. TFOS DEWS II Diagnostic Methodology Report recommends performing NIBUT. NIBUT uses infrared light to detect the teat break up time [70,71].
- Meniscometry: The height of the tear film meniscus can represent the amount of tear production. The meniscus can be measured by either slit lamp examination or optical coherence tomography [42]. The normal tear meniscus height ranges from 0.1 mm to 0.25 mm [72].
- Meibomian Glands: When the Meibomian glands are clogged or inflamed, the tear film lacks an adequate oil layer. [73,74]. Microscopic examination of the Meibomian glands can be performed under slit lamp. Noncontact infrared meibography allows for the evaluation and grading of the morphological changes in the meibomian glands [74].
- Lid Parallel Conjunctival Folds (LIPCOF): LIPCOF are the folds on the lateral temporal conjunctiva near the inferior fornix. An increased number of LIPCOFs is indicative of DED [75].
- Ocular Surface Staining: Staining of the ocular surface can be helpful in assessing the severity of DED [76]. For corneal staining, fluorescein is commonly used, as it stains anywhere there is epithelial damage [77]. For conjunctival staining, lissamine green can be used to stain epithelium that lacks a mucous coating and dead cells. Rose Bengal is not recommended due to its cytotoxicity [78]. Lissamine green was better tolerated than Rose Bengal among patients with DED as well [79].

## 6. Management

### 6.1. Management by PCPs

**Figure 4:**
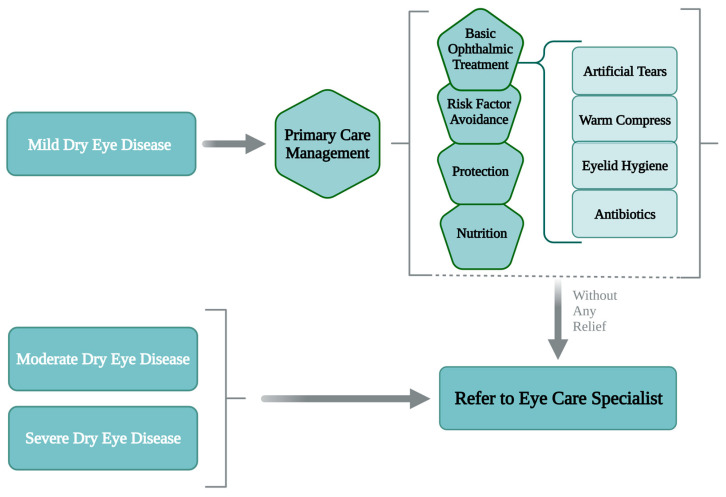
Management of mild dry eye disease in primary care setting.

DED can be managed in a primary care setting through basic ophthalmic treatment, risk factor avoidance, protection, and nutritional support (Figure 4). The methods described here are most effective for mild dry eye conditions. However, moderate to severe conditions require additional specialist visit and advanced treatment [80].

#### 6.1.1. Basic Ophthalmic Treatment

Artificial lubricants, warm compresses, and lid hygiene are palliative measures that help moisturize the eyes [81,82].

**Figure 5:**
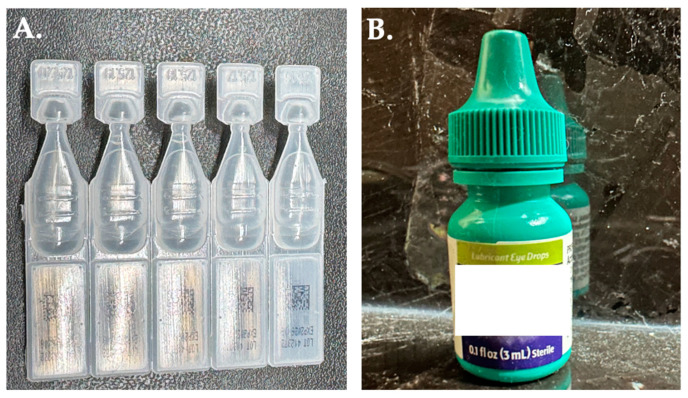
Comparison of artificial tears (**A**) without preservatives (**B**) with preservatives. (**A**). Preservative-free artificial tears are typically packaged in single-use vials. (**B**) The multi-dose preservative artificial tears are packaged in an eye drop bottle.

- Preservative-Free Artificial Tears: Using artificial drops is the first step in DED management. Constant and long-term application of artificial drops four times daily can decrease severity of DED. For patients with evaporation dry eye condition, drops containing liposomes are more effective [83]. Lubricants could be based on tear drops, gels, and ointments [80]. Gels and ointments are more viscous than drops, providing greater stability and retention on the ocular surface; however, they can blur vision and are therefore recommended for nighttime use [80]. Of note, it is important to use single-use preservative-free artificial tears rather than bottled ones with preservatives (Figure 5). Preservatives are toxic to the ocular surface and can worsen dry eye [84]. Benzalkonium chloride (BAK), the most frequently used preservative in ophthalmic solution formulation, has dose-dependent toxicity, disrupting the lipid and mucin layer of the tear film and worsening dry eye [85].
- Warm Compress: Warm compresses can alleviate MGD and promote meibum secretion [34]. This helps maintain the healthy lipid layer, which is essential in preventing excessive tear evaporation.
- Eyelid Hygiene: Proper eyelid hygiene is another way to manage MGD and blepharitis. Managing MGD and blepharitis can simultaneously help reduce inflammation and DED. For therapeutic effects, particularly in treating Demodex infestations, cleansers with anti-inflammatory properties, such as tea tree oils, are recommended [86,87]. Clinicians should demonstrate and educate patients on effective at-home cleaning techniques, including the correct use of finger massaging, cotton swabs, cleaning wipes, or lid brushes [86]. Early intervention for blepharitis is essential before it becomes chronic, which could lead to biofilm formation and worsening ocular surface damage [88].
- Antibiotics: Antibiotics such as doxycycline, minocycline, or azithromycin also serve as treatment options for MGD and blepharitis due to their anti-inflammatory and antimicrobial properties [89,90]. They help disrupt the cycle of meibum dysfunction, bacterial infection, inflammation, and tear film instability [90].

It is important to note that topical anesthetics should not be used to treat DED. Topical anesthetics acquired via PCP prescriptions, attempts to self-medicate in unregulated communities, or illegal purchase from pharmacies can lead to the misuse of topical anesthetics in DED [91]. However, the abuse of topical anesthetics is toxic to ocular surfaces with corneal epithelial erosions and worsens dry eye by decreasing blinking and tear production from lack of corneal sensation [92]. Even though topical nonsteroidal anti-inflammatory drugs (NSAIDs) may give temporary pain relief, they damage the corneal epithelium further by decreasing corneal sensation [93,94].

#### 6.1.2. Avoidance of Risk Factors

One of the most important extrinsic factors for DED is digital screen usage (e.g., laptops, tablets, and smartphones). The prevalence of DED among workers using visual display terminals was reported to range from 9.5% to 87.5% [95]. This correlation is hypothesized to primarily result from disruptions in blinking patterns, such as reduced blink rate and incomplete blinking [96]. Focusing on an object 20 feet away for 20 s every 20 min and active blinking can increase ocular tear secretion and improve ocular surface stability [47,97]. Contact lens use, smoking and tobacco, environmental work exposure (e.g., organic solvents in the dry-cleaning industry), low temperatures, air conditioner usage, and insufficient sleep are other contributing factors [98,99,100,101]. Climate, dry seasons, and windy weather can affect dry eyes [102,103]. Certain medications can induce DED, including antihistamines, antihypertensives, antipsychotics, antidepressants, diuretics, oral steroids, and anti-glaucoma [104,105]. Frederick et al. provides a comprehensive list of medications that can cause DED as a side effect [104]. When PCPs prescribe these medications, they should reconsider the drug of choice if the patient has concomitant DED or develops DED as a side effect. PCPs can encourage lifestyle modifications and risk factor avoidance as a primary approach to reducing dry eye deterioration.

#### 6.1.3. Protection and Prevention

Implementing breaks during digital device use, such as following the 20-20-20 rule (i.e., looking 20 feet away for 20 s after every 20 min of continuous screen use), was shown as an effective strategy for reducing dry eye symptoms [106,107,108,109]. Software programs like “eyeblink“ (https://www.blinkingmatters.com/, accessed on 2 January 2025) can provide reminders to take breaks and increase blinking frequency. Using sunglasses in windy areas and moisture chamber eyewear in the workplace can provide effective protection against DED [110]. Increasing the humidity of the local environment through the use of humidifiers and similar devices have shown potential to alleviate DED symptoms as well [111,112].

#### 6.1.4. Nutritional Support

Essential fatty acids (EFAs), such as ω-6 and ω-3, play a vital role in maintaining cell membrane integrity, supporting cell development, and promoting the growth of the nervous system [113]. Additionally, the anti-inflammatory effects of EFAs can help with DED [114]. Vitamins play essential roles in vision and the nervous system. Vitamin C and E have antioxidative properties that prevent ocular surface damage, protecting against DED [115]. Vitamin A plays a key role in stabilizing the ocular surface by contributing to the formation of mucin in the tear film [115]. Lean beef and eggs are rich sources of vitamin A [116].

### 6.2. Management by Eye Care Specialists After Referral

Mild DED can be managed by PCPs with the approaches mentioned above. PCPs can also treat mild DED through artificial tears and basic antibiotics [80]. Moderate or severe DED, however, requires a visit to an optometrist or ophthalmologist and more advanced medications and interventions [80]. Below is a summary of interventions by eye care specialists, including non-surgical treatments, surgical treatments, and devices.

- Cyclosporine: Cyclosporine A inhibits T cells and the subsequent release of cytokines. It is effective in patients with less severe conditions whose symptoms are not alleviated by primary care methods, such as hygiene, lubrication, and environmental modifications [117]. It also promotes conjunctival cell protection through its anti-apoptotic effects and stimulates goblet cell proliferation [117].
- Lifitegrast: An FDA-approved ophthalmic drop inhibits interactions between ICAM-1 and lymphocyte function-associated antigen-1. A Phase III clinical trial involving 711 participants demonstrated a significant improvement in the Eye Dryness Score on day 84 among the treatment group compared to the control group (treatment effect [TE]: 7.16; 95% confidence interval [CI]: 3.04–11.28; *p* = 0.0007) [118]. Long-term safety was confirmed with no opportunistic infections or immunosuppression were observed [119].
- Autologous Serum: Serum is the liquid remnant of blood after coagulation. Serum has the following factors and nutrients that can promote epithelial improvement: albumin, lactoferrin, immunoglobulins, vitamin A, transforming growth factor-β (TGF-β), fibronectin, epithelial growth factor, and basic fibroblast growth factor [120,121]. Although drawbacks like contamination risks, reliance on the patient’s blood, and the gradual inactivation of components over time are not ideal, their effectiveness has driven scientists to develop more advanced and efficient tear substitutes [120].
- Corticosteroid: Different trials comparing corticosteroid alone, corticosteroid combined with tobramycin versus artificial tears, or artificial tears with tobramycin and no treatment showed minor to moderate symptom improvement with a standardized mean difference of 0.29 [122] but with no evidence in improving tear film quality or quantity [122].
- Scleral Contact Lenses (SCL): SCL covers not only the cornea but also the sclera. It creates the reservoir of tears that keeps the ocular surface moisturized. SCL is a gas permeable yet rigid lens that creates an extra protective layer against the cornea from the external environment [123]. While current evidence is insufficient to recommend SCL for DED patients without concurrent corneal diseases, physicians are still offering SCLs due to their many potential benefits [124]. Studies have also reported significant improvements in OSDI scores, reduced tear osmolarity, and improvement in corneal and conjunctival staining in DED patients with associated corneal irregularities or other forms of ocular surface diseases who were fitted with SCLs [124,125].
- Punctal Occlusion: Punctal plugs can be used to occlude the tear drainage system to maintain the tear film [126]. Laser or cautery can be used to permanently occlude the puncta. Eleven patients underwent permanent punctal occlusion, and after approximately one year, 64% showed alleviation of their symptoms [127]. Punctal occlusion is typically used for patients with moderate to severe dry eye disease refractory to medical treatments [128].
- Tarsorrhaphy: Tarsorrhaphy closes the eyelids partially or completely to reduce tear evaporation [129]. When covered by the eyelid mechanically, the cornea is constantly relubricated every time the eye moves [130].
- Amniotic Membrane: Amniotic membrane has anti-inflammatory, regenerative, and anti-scarring properties [131]. Amniotic membrane can be applied beneath a contact lens as a non-surgical intervention [132]. Amniotic membrane transplantation (AMT) is also a surgical treatment option for DED. A study showed that the corneal sensation of the participants with severe DED who received cryopreserved AMT increased from 3.25 ± 0.6 cm at baseline to 5.6 ± 0.4 cm at the 3-month follow-up (*p* < 0.001) [133].
- Light-based, Heat-based, and Other New Technology: Intense Pulsed Light (IPL) targets vascular and pigmented cells, converting absorbed light into destructive heat. It is often used for EDE, particularly in cases of rosacea. IPL emits light at a wavelength of 500 nm, reducing ocular inflammation, bacterial overgrowth, and meibomian gland obstruction [134]. Low-level light therapy (LLLT), or photobiomodulation, is another light-based therapy utilizing near-infrared or red light to treat DED [135]. While studies on the efficacy of LLLT as a stand-alone treatment remain limited, Antwi et al. recently reported significant improvements in tear film stability and ocular comfort in patients with mild to moderate DED following three LLLT session over 3 weeks [132,135]. Vectored Thermal Pulsation (LipiFlow™) is a heat-based therapy that delivers repetitive, graded heat and compression to the conjunctiva and meibomian glands, helping to reduce obstruction [136,137]. MiBo Thermoflo, another heat-based treatment, applies a thermoelectric heat probe to the eyelids to enhance meibomian gland secretion and tear film quality [138]. The Intranasal Tear Neurostimulator (TrueTear^®^), an FDA-approved treatment for DED, is designed to stimulate the nerves, supplying the lacrimal functional unit to increase the tear production [139].

## 7. Conclusions

DED is an ocular surface disease characterized by the loss of tear film homeostasis, presenting with a spectrum of clinical symptoms ranging from mild to severe. Despite being a leading cause of ophthalmologic consultations, DED remains underdiagnosed in primary care settings. This review highlights tools and strategies to help PCPs enhance their diagnostic and management capabilities, equipping them with an improved understanding of DED’s epidemiology, pathophysiology, and clinical presentations. However, a limitation is that many diagnostic criteria still require specialized diagnostic equipment, which may be inaccessible to many PCPs or necessitate an ophthalmologist’s slit lamp examination. Tailoring distinct DED evaluation guidelines for PCPs and tests that are easily accessible in the primary care setting would be beneficial, especially for patients who have limited access to healthcare. Emerging technologies, such as artificial intelligence (AI), show promise in addressing this diagnostic gap in the future. Heidari et al. demonstrated the potential of AI-based models in DED diagnosis using various imaging modalities (e.g., keratography, meibography, anterior segment optical coherence tomography) to achieve an overall accuracy of 91.91% [140]. By facilitating earlier detection and screening, especially in underserved areas, AI and similar emerging tools can enable PCPs to identify and manage DED more effectively, thereby mitigating disease progression and improving patient outcomes.

## Figures and Tables

**Table 1:** Systemic disease association with DED from multiple studies [48,49,50,51].

Disease	OR	95% CI	*p*-Value
Sjögren’s syndrome [50]	60.3	27.0–135	<0.001
Graves’ disease [50]	4.58	3.22–6.50	<0.001
Systemic lupus erythematous [50]	4.21	2.09–8.51	<0.001
Systemic sclerosis [50]	2.96	1.35–6.50	0.007
Depression [48]	2.92	2.13–4.01	<0.00001
Anxiety [48]	2.80	2.61–3.02	<0.00001
Fibromyalgia [50]	2.21	2.03–2.41	<0.001
Crohn [50]	2.01	1.51–2.70	<0.001
Rosacea [50]	1.95	1.28–2.97	0.002
Sarcoidosis [50]	1.94	1.43–2.65	<0.001
Rheumatoid arthritis [50]	1.94	1.76–2.15	<0.001
ADHD [50]	1.93	1.52–2.45	<0.0001
Ankylosing spondylitis [50]	1.74	1.09–2.78	0.02
Thyroid disease [51]	1.41	1.09–1.84	<0.01
Diabetes Mellitus [49]	1.30	1.08–1.57	0.006

Note: Data are derived from multiple sources with different patient populations.

**Table 2:** OSDI questionnaire.

	All the Time	Most of the Time	Half of the Time	Some of the Time	None of the Time
Have you experienced any of the following during the last week:					
Eyes that are sensitive to light?	4	3	2	1	0
2. Eyes that feel gritty?	4	3	2	1	0
3. Painful or sore eyes?	4	3	2	1	0
4. Blurred vision?	4	3	2	1	0
5. Poor vision?	4	3	2	1	0
Have problems with your eyes limited you in performing any of the following during the last week:					
6. Reading?	4	3	2	1	0
7. Driving at night?	4	3	2	1	0
8. Working with a computer or bank machine (ATM)?	4	3	2	1	0
9. Watching TV?	4	3	2	1	0
Have your eyes felt uncomfortable in any of the following situations during the last week:					
10. Windy conditions?	4	3	2	1	0
11. Places or areas with low humidity (very dry)?	4	3	2	1	0
12. Areas that are air conditioned?	4	3	2	1	0

**Table 3:** DEQ-5 questionnaire.

	Never	Rarely	Sometimes	Frequently	Constantly
Questions about Eye Discomfort:					
a. During a typical day in the past month, how often did your eyes feel discomfort?	0	1	2	3	4
b. When your eyes feel discomfort, how intense was this feeling of discomfort at the end of the day, within two hours of going to bed?	0	1	2	3	4
2. Questions about Eye Dryness:					
a. During a typical day in the past month, how often did your eyes feel dry?	0	1	2	3	4
b. When your eyes felt dry, how intense was this feeling of dryness at the end of the day, within two hours of going to bed?	0	1	2	3	4
3. Question about Watery Eyes					
a. During a typical day in the past month, how often did your eyes look or feel excessively watery?	0	1	2	3	4

## Data Availability

No new data were created.

## Abbreviations

The following abbreviations are used in this manuscript:

DED	dry eye disease
PCP	primary care physicians
ADDE	aqueous-deficient dry eye
EDE	evaporative dry eye
TFOS	Tear Film and Ocular Surface Society
DEWS	Dry Eye Workshop
SSDE	Sjögren Syndrome Dry Eye
NSDE	Non-Sjögren Syndrome Dry Eye
MGD	meibomian gland dysfunction
TED	thyroid eye disease
LASIK	laser-assisted in situ keratomileusis
SMILE	small incision lenticule extraction
PRK	photorefractive keratectomy
DEQ-5	Dry Eye Questionnaire
OMMP	Ocular Mucous Membrane Pemphigoid.
GVHD	Graft-Versus-Host Disease
OSDI	Ocular Surface Disease Index
NIBUT	Non-Invasive Breakup Time
MMP-9	Matrix Metalloproteinase-9
NICE	National Institute for Health and Care Excellence
LIPCOF	Lid Parallel Conjunctival Folds
BAK	benzalkonium chloride
NSAIDs	nonsteroidal anti-inflammatory drugs
EFAs	essential fatty acids
TGF-β	transforming growth factor-β
SCL	Scleral Contact Lenses
AMT	amniotic membrane transplantation
IPL	Intense Pulsed Light
LLLT	Low-Level Light Therapy
AI	artificial intelligence

## References

[1] Craig J.P., Nichols K.K., Akpek E.K., Caffery B., Dua H.S., Joo C.-K., Liu Z., Nelson J.D., Nichols J.J., Tsubota K. (2017). TFOS DEWS II Definition and Classification Report. Ocul. Surf..

[2] Akowuah P.K., Kobia-Acquah E. (2020). Prevalence of Dry Eye Disease in Africa: A Systematic Review and Meta-Analysis. Optom. Vis. Sci..

[3] Cai Y., Wei J., Zhou J., Zou W. (2022). Prevalence and Incidence of Dry Eye Disease in Asia: A Systematic Review and Meta-Analysis. Ophthalmic Res..

[4] McCann P., Abraham A.G., Mukhopadhyay A., Panagiotopoulou K., Chen H., Rittiphairoj T., Gregory D.G., Hauswirth S.G., Ifantides C., Qureshi R. (2022). Prevalence and Incidence of Dry Eye and Meibomian Gland Dysfunction in the United States: A Systematic Review and Meta-Analysis. JAMA Ophthalmol..

[5] Stapleton F., Alves M., Bunya V.Y., Jalbert I., Lekhanont K., Malet F., Na K.-S., Schaumberg D., Uchino M., Vehof J. (2017). TFOS DEWS II Epidemiology Report. Ocul. Surf..

[6] Papas E.B. (2021). The Global Prevalence of Dry Eye Disease: A Bayesian View. Ophthalmic Physiol. Opt..

[7] Benítez-del-Castillo J.M., Burgos-Blasco B. (2025). Prevalence of Dry Eye Disease in Spain: A Population-Based Survey (PrevEOS). Ocul. Surf..

[8] Zhang Y., Chen H., Wu X. (2012). Prevalence and Risk Factors Associated with Dry Eye Syndrome among Senior High School Students in a County of Shandong Province, China. Ophthalmic Epidemiol..

[9] Uchino M., Dogru M., Uchino Y., Fukagawa K., Shimmura S., Takebayashi T., Schaumberg D.A., Tsubota K. (2008). Japan Ministry of Health Study on Prevalence of Dry Eye Disease Among Japanese High School Students. Am. J. Ophthalmol..

[10] Stapleton F., Velez F.G., Lau C., Wolffsohn J.S. (2024). Dry Eye Disease in the Young: A Narrative Review. Ocul. Surf..

[11] Kim J.S., Wang M.T.M., Craig J.P. (2019). Exploring the Asian Ethnic Predisposition to Dry Eye Disease in a Pediatric Population. Ocul. Surf..

[12] Wang M.T.M., Craig J.P., Vidal-Rohr M., Menduni F., Dhallu S., Ipek T., Acar D., Recchioni A., France A., Kingsnorth A. (2022). Impact of Digital Screen Use and Lifestyle Factors on Dry Eye Disease in the Paediatric Population. Ocul. Surf..

[13] Moon J.H., Kim K.W., Moon N.J. (2016). Smartphone Use Is a Risk Factor for Pediatric Dry Eye Disease According to Region and Age: A Case Control Study. BMC Ophthalmol..

[14] Ma J., Zhu H., Guo W., Li R., Shen S., Wang Y., Huang D., Zhang X., Fu Z., Zhao A. (2022). Association of Different Digital Media Experiences with Paediatric Dry Eye in China: A Population-Based Study. BMJ Open.

[15] Wolffsohn J.S., Wang M.T.M., Vidal-Rohr M., Menduni F., Dhallu S., Ipek T., Acar D., Recchioni A., France A., Kingsnorth A. (2021). Demographic and Lifestyle Risk Factors of Dry Eye Disease Subtypes: A Cross-Sectional Study. Ocul. Surf..

[16] Qian L., Wei W. (2022). Identified Risk Factors for Dry Eye Syndrome: A Systematic Review and Meta-Analysis. PLoS ONE.

[17] Wang M.T.M., Vidal-Rohr M., Muntz A., Diprose W.K., Ormonde S.E., Wolffsohn J.S., Craig J.P. (2020). Systemic Risk Factors of Dry Eye Disease Subtypes: A New Zealand Cross-Sectional Study. Ocul. Surf..

[18] Rolando M., Zierhut M. (2001). The Ocular Surface and Tear Film and Their Dysfunction in Dry Eye Disease. Surv. Ophthalmol..

[19] Willcox M.D.P., Argüeso P., Georgiev G.A., Holopainen J.M., Laurie G.W., Millar T.J., Papas E.B., Rolland J.P., Schmidt T.A., Stahl U. (2017). TFOS DEWS II Tear Film Report. Ocul. Surf..

[20] Wolff E. (1946). The Mucocutaneous Junction of the Lidmargin and the Distribution of the Tear Fluid. Trans. Ophthalmol. Soc. UK.

[21] Bron A.J., De Paiva C.S., Chauhan S.K., Bonini S., Gabison E.E., Jain S., Knop E., Markoulli M., Ogawa Y., Perez V. (2017). TFOS DEWS II Pathophysiology Report. Ocul. Surf..

[22] Hakim F.E., Farooq A.V. (2022). Dry Eye Disease: An Update in 2022. JAMA.

[23] Coursey T.G., de Paiva C.S. (2014). Managing Sjögren’s Syndrome and Non-Sjögren Syndrome Dry Eye with Anti-Inflammatory Therapy. Clin. Ophthalmol..

[24] Pflugfelder S.C., Jones D., Ji Z., Afonso A., Monroy D. (1999). Altered Cytokine Balance in the Tear Fluid and Conjunctiva of Patients with Sjögren’s Syndrome Keratoconjunctivitis Sicca. Curr. Eye Res..

[25] Golden M.I., Meyer J.J., Zeppieri M., Patel B.C. (2024). Dry Eye Syndrome. StatPearls.

[26] Sheppard J., Shen Lee B., Periman L.M. (2023). Dry Eye Disease: Identification and Therapeutic Strategies for Primary Care Clinicians and Clinical Specialists. Ann. Med..

[27] Zhang X., Zhao L., Deng S., Sun X., Wang N. (2016). Dry Eye Syndrome in Patients with Diabetes Mellitus: Prevalence, Etiology, and Clinical Characteristics. J. Ophthalmol..

[28] Ziaragkali S., Kotsalidou A., Trakos N. (2018). Dry Eye Disease in Routine Rheumatology Practice. Mediterr. J. Rheumatol..

[29] Rana H.S., Akella S.S., Clabeaux C.E., Skurski Z.P., Aakalu V.K. (2022). Ocular Surface Disease in Thyroid Eye Disease: A Narrative Review. Ocul. Surf..

[30] Doughty M.J. (2011). Contact Lens Wear and the Goblet Cells of the Human Conjunctiva—A Review. Contact Lens Anterior Eye.

[31] Huang R., Su C., Fang L., Lu J., Chen J., Ding Y. (2022). Dry Eye Syndrome: Comprehensive Etiologies and Recent Clinical Trials. Int. Ophthalmol..

[32] Sommer A., Emran N., Tamba T. (1979). Vitamin A-Responsive Punctate Keratopathy in Xerophthalmia. Am. J. Ophthalmol..

[33] Sullivan D.A. (2004). Sex and Sex Steroid Influences on Dry Eye Syndrome. Dry Eye and Ocular Surface Disorders.

[34] Narang P., Donthineni P.R., D’Souza S., Basu S. (2023). Evaporative Dry Eye Disease Due to Meibomian Gland Dysfunction: Preferred Practice Pattern Guidelines for Diagnosis and Treatment. Indian J. Ophthalmol..

[35] Rolando M., Merayo-Lloves J. (2022). Management Strategies for Evaporative Dry Eye Disease and Future Perspective. Curr. Eye Res..

[36] Zeev M.S.-B., Miller D.D., Latkany R. (2014). Diagnosis of Dry Eye Disease and Emerging Technologies. Clin. Ophthalmol..

[37] Tsubota K., Yokoi N., Shimazaki J., Watanabe H., Dogru M., Yamada M., Kinoshita S., Kim H.-M., Tchah H.-W., Hyon J.Y. (2017). New Perspectives on Dry Eye Definition and Diagnosis: A Consensus Report by the Asia Dry Eye Society. Ocul. Surf..

[38] Akpek E.K., Amescua G., Farid M., Garcia-Ferrer F.J., Lin A., Rhee M.K., Varu D.M., Musch D.C., Dunn S.P., Mah F.S. (2019). Dry Eye Syndrome Preferred Practice Pattern^®^. Ophthalmology.

[39] Lira M., Oliveira M.E.C.R., Franco S. (2011). Comparison of the Tear Film Clinical Parameters at Two Different Times of the Day. Clin. Exp. Optom..

[40] Mohamed H.B., Abd El-Hamid B.N., Fathalla D., Fouad E.A. (2022). Current Trends in Pharmaceutical Treatment of Dry Eye Disease: A Review. Eur. J. Pharm. Sci..

[41] Nair S., Kaur M., Sharma N., Titiyal J.S. (2023). Refractive Surgery and Dry Eye—An Update. Indian J. Ophthalmol..

[42] Messmer E.M. (2015). The Pathophysiology, Diagnosis, and Treatment of Dry Eye Disease. Dtsch. Arzteblatt Int..

[43] Labetoulle M., Bourcier T., Doan S. (2019). Classifying Signs and Symptoms of Dry Eye Disease According to Underlying Mechanism via the Delphi Method: The DIDACTIC Study. Br. J. Ophthalmol..

[44] Donthineni P.R., Doctor M.B., Shanbhag S., Kate A., Galor A., Djalilian A.R., Singh S., Basu S. (2023). Aqueous-Deficient Dry Eye Disease: Preferred Practice Pattern Guidelines on Clinical Approach, Diagnosis, and Management. Indian J. Ophthalmol..

[45] Findlay Q., Reid K. (2018). Dry Eye Disease: When to Treat and When to Refer. Aust. Prescr..

[46] Wolffsohn J.S., Arita R., Chalmers R., Djalilian A., Dogru M., Dumbleton K., Gupta P.K., Karpecki P., Lazreg S., Pult H. (2017). TFOS DEWS II Diagnostic Methodology Report. Ocul. Surf..

[47] Verjee M.A., Brissette A.R., Starr C.E. (2020). Dry Eye Disease: Early Recognition with Guidance on Management and Treatment for Primary Care Family Physicians. Ophthalmol. Ther..

[48] Wan K.H., Chen L.J., Young A.L. (2016). Depression and Anxiety in Dry Eye Disease: A Systematic Review and Meta-Analysis. Eye.

[49] Yoo T.K., Oh E. (2019). Diabetes Mellitus Is Associated with Dry Eye Syndrome: A Meta-Analysis. Int. Ophthalmol..

[50] Vehof J., Snieder H., Jansonius N., Hammond C.J. (2021). Prevalence and Risk Factors of Dry Eye in 79,866 Participants of the Population-Based Lifelines Cohort Study in the Netherlands. Ocul. Surf..

[51] Moss S.E., Klein R., Klein B.E.K. (2000). Prevalence of and Risk Factors for Dry Eye Syndrome. Arch. Ophthalmol..

[52] Schiffman R.M., Christianson M.D., Jacobsen G., Hirsch J.D., Reis B.L. (2000). Reliability and Validity of the Ocular Surface Disease Index. Arch. Ophthalmol..

[53] Grubbs J.R., Tolleson-Rinehart S., Huynh K., Davis R.M. (2014). A Review of Quality of Life Measures in Dry Eye Questionnaires. Cornea.

[54] Abetz L., Rajagopalan K., Mertzanis P., Begley C., Barnes R., Chalmers R. (2011). Development and Validation of the Impact of Dry Eye on Everyday Life (Ideel) Questionnaire, a Patient-Reported Outcomes (pro) Measure for the Assessment of the Burden of Dry Eye on Patients. Health Qual. Life Outcomes.

[55] Chalmers R.L., Begley C.G., Caffery B. (2010). Validation of the 5-Item Dry Eye Questionnaire (DEQ-5): Discrimination across Self-Assessed Severity and Aqueous Tear Deficient Dry Eye Diagnoses. Contact Lens Anterior Eye.

[56] Thulasi P., Djalilian A. (2017). Update in Current Diagnostics and Therapeutics of Dry Eye Disease. Ophthalmology.

[57] Johnston P.R., Rodriguez J., Lane K.J., Ousler G., Abelson M.B. (2013). The Interblink Interval in Normal and Dry Eye Subjects. Clin. Ophthalmol..

[58] Wolffsohn J.S., Craig J.P., Vidal-Rohr M., Huarte S.T., Ah Kit L., Wang M. (2018). Blink Test Enhances Ability to Screen for Dry Eye Disease. Contact Lens Anterior Eye.

[59] Bron A.J., Argüeso P., Irkec M., Bright F.V. (2015). Clinical Staining of the Ocular Surface: Mechanisms and Interpretations. Prog. Retin. Eye Res..

[60] Kaur S., Larsen H., Nattis A. (2019). Primary Care Approach to Eye Conditions. Osteopath. Fam. Physician.

[61] van Tilborg M.M.A., Murphy P.J., Evans K.S.E. (2015). Agreement in Dry Eye Management Between Optometrists and General Practitioners in Primary Health Care in the Netherlands. Contact Lens Anterior Eye.

[62] Savini G., Prabhawasat P., Kojima T., Grueterich M., Espana E., Goto E. (2008). The Challenge of Dry Eye Diagnosis. Clin. Ophthalmol..

[63] Sade De Paiva C., Pflugfelder S.C. (2004). Corneal Epitheliopathy of Dry Eye Induces Hyperesthesia to Mechanical Air Jet Stimulation. Am. J. Ophthalmol..

[64] Spierer O., Felix E.R., McClellan A.L., Parel J.M., Gonzalez A., Feuer W.J., Sarantopoulos C.D., Levitt R.C., Ehrmann K., Galor A. (2016). Corneal Mechanical Thresholds Negatively Associate with Dry Eye and Ocular Pain Symptoms. Investig. Ophthalmol. Vis. Sci..

[65] Bourcier T., Acosta M.C., Borderie V., Borrás F., Gallar J., Bury T., Laroche L., Belmonte C. (2005). Decreased Corneal Sensitivity in Patients with Dry Eye. Investig. Ophthalmol. Vis. Sci..

[66] Benítez-del-Castillo J.M., Acosta M.C., Wassfi M.A., Díaz-Valle D., Gegúndez J.A., Fernandez C., García-Sánchez J. (2007). Relation between Corneal Innervation with Confocal Microscopy and Corneal Sensitivity with Noncontact Esthesiometry in Patients with Dry Eye. Investig. Ophthalmol. Vis. Sci..

[67] Bonini S., Rama P., Olzi D., Lambiase A. (2003). Neurotrophic Keratitis. Eye.

[68] Schultheis W.G., Hampton T., Gensheimer W. (2024). Comparison of Cochet–Bonnet, Dental Floss, and Cotton Wisp with Applications to Corneal Sensation Testing. Cornea.

[69] Patel S., Mehra D., Cabrera K., Galor A. (2021). How Should Corneal Nerves Be Incorporated into the Diagnosis and Management of Dry Eye?. Curr. Ophthalmol. Rep..

[70] Muhafiz E., Demir M.S. (2022). Ability of Non-Invasive Tear Break-up Time to Determine Tear Instability in Contact Lens Wearers. Int. Ophthalmol..

[71] Mengher L.S., Bron A.J., Tonge S.R., Gilbert D.J. (1985). A Non-Invasive Instrument for Clinical Assessment of the Pre-Corneal Tear Film Stability. Curr. Eye Res..

[72] Doughty M.J., Laiquzzaman M., Oblak E., Button N. (2002). The Tear (Lacrimal) Meniscus Height in Human Eyes: A Useful Clinical Measure or an Unusable Variable Sign?. Contact Lens Anterior Eye.

[73] Chhadva P., Goldhardt R., Galor A. (2017). Meibomian Gland Disease: The Role of Gland Dysfunction in Dry Eye Disease. Ophthalmology.

[74] Arita R., Itoh K., Inoue K., Amano S. (2008). Noncontact Infrared Meibography to Document Age-Related Changes of the Meibomian Glands in a Normal Population. Ophthalmology.

[75] Pult H., Purslow C., Murphy P.J. (2011). The Relationship Between Clinical Signs and Dry Eye Symptoms. Eye.

[76] Craig J.P., Singh I., Tomlinson A., Morgan P.B., Efron N. (2000). The Role of Tear Physiology in Ocular Surface Temperature. Eye.

[77] Feenstra R.P.G., Tseng S.C.G. (1992). Comparison of Fluorescein and Rose Bengal Staining. Ophthalmology.

[78] Lee Y.C., Park C.K., Kim M.S., Kim J.H. (1996). In Vitro Study for Staining and Toxicity of Rose Bengal on Cultured Bovine Corneal Endothelial Cells. Cornea.

[79] Hamrah P., Alipour F., Jiang S., Sohn J.-H., Foulks G.N. (2011). Optimizing Evaluation of Lissamine Green Parameters for Ocular Surface Staining. Eye.

[80] Safir M., Twig G., Mimouni M. (2024). Dry Eye Disease Management. BMJ.

[81] Sung J., Wang M.T.M., Lee S.H., Cheung I.M.Y., Ismail S., Sherwin T., Craig J.P. (2018). Randomized Double-Masked Trial of Eyelid Cleansing Treatments for Blepharitis. Ocul. Surf..

[82] Lee G. (2024). Evidence-Based Strategies for Warm Compress Therapy in Meibomian Gland Dysfunction. Ophthalmol. Ther..

[83] Semp D.A., Beeson D., Sheppard A.L., Dutta D., Wolffsohn J.S. (2023). Artificial Tears: A Systematic Review. Clin. Optom..

[84] Albietz J.M., Bruce A.S. (2001). The Conjunctival Epithelium in Dry Eye Subtypes: Effect of Preserved and Non-Preserved Topical Treatments. Curr. Eye Res..

[85] Coroi M.C., Bungau S., Tit M. (2015). Preservatives from the Eye Drops and the Ocular Surface. Rom. J. Ophthalmol..

[86] Zhang L., Wang J., Gao Y. (2023). Eyelid Cleaning: Methods, Tools, and Clinical Applications. Indian J. Ophthalmol..

[87] Koo H., Kim T.H., Kim K.W., Wee S.W., Chun Y.S., Kim J.C. (2012). Ocular Surface Discomfort and *Demodex*: Effect of Tea Tree Oil Eyelid Scrub in *Demodex* Blepharitis. J. Korean Med. Sci..

[88] Rynerson J., Perry H. (2016). Debs—A Unification Theory for Dry Eye and Blepharitis. Clin. Ophthalmol..

[89] Frucht-Pery J., Sagi E., Hemo I., Ever-Hadani P. (1993). Efficacy of Doxycycline and Tetracycline in Ocular Rosacea. Am. J. Ophthalmol..

[90] Vernhardsdottir R.R., Magno M.S., Hynnekleiv L., Lagali N., Dartt D.A., Vehof J., Jackson C.J., Utheim T.P. (2022). Antibiotic Treatment for Dry Eye Disease Related to Meibomian Gland Dysfunction and Blepharitis—A Review. Ocul. Surf..

[91] Sharifi A., Naisiri N., Shams M., Sharifi M., Sharifi H. (2022). Adverse Reactions from Topical Ophthalmic Anesthetic Abuse. J. Ophthalmic Vis. Res..

[92] McGee H.T., Fraunfelder F.W. (2007). Toxicities of Topical Ophthalmic Anesthetics. Expert Opin. Drug Saf..

[93] Singer D.D., Kennedy J., Wittpenn J.R. (2015). Topical NSAIDs Effect on Corneal Sensitivity. Cornea.

[94] Nguyen A., Kolluru A., Beglarian T. (2023). Dry Eye Disease: A Review of Anti-Inflammatory Therapies. Taiwan J. Ophthalmol..

[95] Courtin R., Pereira B., Naughton G., Chamoux A., Chiambaretta F., Lanhers C., Dutheil F. (2016). Prevalence of Dry Eye Disease in Visual Display Terminal Workers: A Systematic Review and Meta-Analysis. BMJ Open.

[96] Al-Mohtaseb Z., Schachter S., Shen Lee B., Garlich J., Trattler W. (2021). The Relationship Between Dry Eye Disease and Digital Screen Use. Clin. Ophthalmol..

[97] Lemp M.A. (2008). Advances in Understanding and Managing Dry Eye Disease. Am. J. Ophthalmol..

[98] Alven A., Lema C., Redfern R.L. (2021). Impact of Low Humidity on Damage-Associated Molecular Patterns at the Ocular Surface during Dry Eye Disease. Optom. Vis. Sci..

[99] Sapkota K., Martin R., Franco S., Lira M. (2015). Common Symptoms of Nepalese Soft Contact Lens Wearers: A Pilot Study. J. Optom..

[100] Jiménez Barbosa I.A., Rodríguez Alvarez M.F., Dussán Torres G.A., Khuu S.K. (2019). Ocular Surface and Tear Film Changes in Workers Exposed to Organic Solvents Used in the Dry-Cleaning Industry. PLoS ONE.

[101] Chlasta-Twardzik E., Górecka-Nitoń A., Nowińska A., Wylęgała E. (2021). The Influence of Work Environment Factors on the Ocular Surface in a One-Year Follow-Up Prospective Clinical Study. Diagnostics.

[102] Van Setten G., Labetoulle M., Baudouin C., Rolando M. (2016). Evidence of Seasonality and Effects of Psychrometry in Dry Eye Disease. Acta Ophthalmol..

[103] Alves M., Asbell P., Dogru M., Giannaccare G., Grau A., Gregory D., Kim D.H., Marini M.C., Ngo W., Nowinska A. (2023). TFOS Lifestyle Report: Impact of Environmental Conditions on the Ocular Surface. Ocul. Surf..

[104] Fraunfelder F.T., Sciubba J.J., Mathers W.D. (2012). The Role of Medications in Causing Dry Eye. J. Ophthalmol..

[105] Maeng K.J., Lee K., Kim S., Park C.K., Kim E.W., Lee S.Y., Bae H.W., Seong G.J., Kim C.Y. (2021). Effects of Glaucoma Medication on Dry Eye Syndrome and Quality of Life in Patients with Glaucoma. Korean J. Ophthalmol..

[106] Talens-Estarelles C., Cerviño A., García-Lázaro S., Fogelton A., Sheppard A., Wolffsohn J.S. (2023). The Effects of Breaks on Digital Eye Strain, Dry Eye and Binocular Vision: Testing the 20-20-20 Rule. Contact Lens Anterior Eye.

[107] Alghamdi W.M., Alrasheed S.H. (2020). Impact of an Educational Intervention Using the 20/20/20 Rule on Computer Vision Syndrome. Afr. Vis. Eye Health.

[108] Zulkarnain B.S., Budiyatin A.S., Aryani T., Loebis R. (2021). The Effect of 20-20-20 Rule Dissemination and Artificial Tears Administration in High School Students Diagnosed with Computer Vision Syndrome. Indones J. Commun. Engag..

[109] Nosch D.S., Foppa C., Tóth M., Joos R.E. (2015). Blink Animation Software to Improve Blinking and Dry Eye Symptoms. Optom. Vis. Sci..

[110] Milner M.S., Beckman K.A., Luchs J.I., Allen Q.B., Awdeh R.M., Berdahl J., Boland T.S., Buznego C., Gira J.P., Goldberg D.F. (2017). Dysfunctional Tear Syndrome: Dry Eye Disease and Associated Tear Film Disorders—New Strategies for Diagnosis and Treatment. Curr. Opin. Ophthalmol..

[111] Wang M.T.M., Chan E., Ea L., Kam C., Lu Y., Misra S.L., Craig J.P. (2017). Randomized Trial of Desktop Humidifier for Dry Eye Relief in Computer Users. Optom. Vis. Sci..

[112] Hirayama M., Murat D., Liu Y., Kojima T., Kawakita T., Tsubota K. (2013). Efficacy of a Novel Moist Cool Air Device in Office Workers with Dry Eye Disease. Acta Ophthalmol..

[113] Darios F., Davletov B. (2006). Omega-3 and Omega-6 Fatty Acids Stimulate Cell Membrane Expansion by Acting on Syntaxin 3. Nature.

[114] Bhargava R., Chandra M., Bansal U., Singh D., Ranjan S., Sharma S. (2016). A Randomized Controlled Trial of Omega 3 Fatty Acids in Rosacea Patients with Dry Eye Symptoms. Curr. Eye Res..

[115] Kim J.-M., Choi Y.-J. (2024). Impact of Dietary Nutrients on the Prevalence of Dry Eye Syndrome among Korean Women Aged 40 and above: Evidence from the Korea National Health and Nutrition Examination Survey. Nutrients.

[116] Werner E.R., Haskell M.J., Arnold C.D., Caswell B.L., Iannotti L.L., Lutter C.K., Maleta K.M., Stewart C.P. (2023). The Effects of One Egg Per Day on Vitamin A Status Among Young Malawian Children: A Secondary Analysis of a Randomized Controlled Trial. Curr. Dev. Nutr..

[117] Periman L.M., Mah F.S., Karpecki P.M. (2020). A Review of the Mechanism of Action of Cyclosporine A: The Role of Cyclosporine A in Dry Eye Disease and Recent Formulation Developments. Clin. Ophthalmol..

[118] Holland E.J., Luchs J., Karpecki P.M., Nichols K.K., Jackson M.A., Sall K., Tauber J., Roy M., Raychaudhuri A., Shojaei A. (2017). Lifitegrast for the Treatment of Dry Eye Disease. Ophthalmology.

[119] Donnenfeld E.D., Karpecki P.M., Majmudar P.A., Nichols K.K., Raychaudhuri A., Roy M., Semba C.P. (2016). Safety of Lifitegrast Ophthalmic Solution 5.0% in Patients with Dry Eye Disease: A 1-Year, Multicenter, Randomized, Placebo-Controlled Study. Cornea.

[120] Pan Q., Angelina A., Marrone M., Stark W.J., Akpek E.K. (2017). Autologous Serum Eye Drops for Dry Eye. Cochrane Database Syst. Rev..

[121] Poon A.C., Geerling G., Dart J.K., Fraenkel G.E., Daniels J.T. (2001). Autologous Serum Eyedrops for Dry Eyes and Epithelial Defects: Clinical and in Vitro Toxicity Studies. Br. J. Ophthalmol..

[122] Liu S.-H., Saldanha I.J., Abraham A.G., Rittiphairoj T., Hauswirth S., Gregory D., Ifantides C., Li T. (2022). Topical Corticosteroids for Dry Eye. Cochrane Database Syst. Rev..

[123] Harthan J.S., Shorter E. (2018). Therapeutic Uses of Scleral Contact Lenses for Ocular Surface Disease: Patient Selection and Special Considerations. Clin. Optom..

[124] Qiu S.X., Fadel D., Hui A. (2024). Scleral Lenses for Managing Dry Eye Disease in the Absence of Corneal Irregularities: What Is the Current Evidence?. J. Clin. Med..

[125] La Porta Weber S., Becco De Souza R., Gomes J.Á.P., Hofling-Lima A.L. (2016). The Use of the Esclera Scleral Contact Lens in the Treatment of Moderate to Severe Dry Eye Disease. Am. J. Ophthalmol..

[126] Ervin A., Law A., Pucker A.D. (2017). Punctal Occlusion for Dry Eye Syndrome. Cochrane Database Syst. Rev..

[127] Liu D., Sadhan Y. (2002). Surgical Punctal Occlusion: A Prospective Study. Br. J. Ophthalmol..

[128] Ranjan A., Basu S., Singh S. (2024). Punctal Cautery in Dry Eye Disease: A Systematic Review. Ocul. Surf..

[129] Rb V., Nr B. (2020). Commentary: Tarsorrhaphy: A Stitch in Time. Indian J. Ophthalmol..

[130] Cosar C.B., Cohen E.J., Rapuano C.J., Maus M., Penne R.P., Flanagan J.C., Laibson P.R. (2001). Tarsorrhaphy: Clinical Experience From a Cornea Practice. Cornea.

[131] Tseng S.C.G., Espana E.M., Kawakita T., Di Pascuale M.A., Li W., He H., Liu T.-S., Cho T.-H., Gao Y.-Y., Yeh L.-K. (2004). How Does Amniotic Membrane Work?. Ocul. Surf..

[132] Travé-Huarte S., Wolffsohn J.S. (2024). Bilateral Sutureless Application of Human Dehydrated Amniotic Membrane with a Specialised Bandage Contact Lens for Moderate-to-Severe Dry Eye Disease: A Prospective Study with 1-Month Follow-Up. Clin. Ophthalmol..

[133] John T., Tighe S., Sheha H., Hamrah P., Salem Z.M., Cheng A.M.S., Wang M.X., Rock N.D. (2017). Corneal Nerve Regeneration after Self-Retained Cryopreserved Amniotic Membrane in Dry Eye Disease. J. Ophthalmol..

[134] Toyos R., McGill W., Briscoe D. (2015). Intense Pulsed Light Treatment for Dry Eye Disease Due to Meibomian Gland Dysfunction; A 3-Year Retrospective Study. Photomed. Laser Surg..

[135] Antwi A., Schill A.W., Redfern R., Ritchey E.R. (2024). Effect of Low-Level Light Therapy in Individuals with Dry Eye Disease. Ophthalmic Physiol. Opt. J. Br. Coll. Ophthalmic Opt. Optom..

[136] Malagón-Liceaga A., Recillas-Gispert C., Ruiz-Quintero N.C., Ruelas-Villavicencio A.L. (2023). Treatment of Ocular Rosacea: A Practical Review from an Interdisciplinary Approach. Arch. Soc. Esp. Oftalmol. Engl. Ed..

[137] Greiner J.V. (2012). A Single LipiFlow^®^ Thermal Pulsation System Treatment Improves Meibomian Gland Function and Reduces Dry Eye Symptoms for 9 Months. Curr. Eye Res..

[138] O’Neil E.C., Henderson M., Massaro-Giordano M., Bunya V.Y. (2019). Advances in Dry Eye Disease Treatment. Curr. Opin. Ophthalmol..

[139] Gumus K., Schuetzle K.L., Pflugfelder S.C. (2017). Randomized Controlled Crossover Trial Comparing the Impact of Sham or Intranasal Tear Neurostimulation on Conjunctival Goblet Cell Degranulation. Am. J. Ophthalmol..

[140] Heidari Z., Hashemi H., Sotude D., Ebrahimi-Besheli K., Khabazkhoob M., Soleimani M., Djalilian A.R., Yousefi S. (2024). Applications of Artificial Intelligence in Diagnosis of Dry Eye Disease: A Systematic Review and Meta-Analysis. Cornea.

